# The Psychological Status of General Population in Hubei Province During the COVID-19 Outbreak: A Cross-Sectional Survey Study

**DOI:** 10.3389/fpubh.2021.622762

**Published:** 2021-04-22

**Authors:** Guanmao Chen, Jiaying Gong, Zhangzhang Qi, Shuming Zhong, Ting Su, Jurong Wang, Siying Fu, Li Huang, Ying Wang

**Affiliations:** ^1^Medical Imaging Center, First Affiliated Hospital of Jinan University, Guangzhou, China; ^2^Institute of Molecular and Functional Imaging, Jinan University, Guangzhou, China; ^3^Department of Radiology, Six Affiliated Hospital of Sun Yat-sen University, Guangzhou, China; ^4^Department of Psychiatry, First Affiliated Hospital of Jinan University, Guangzhou, China

**Keywords:** coronavirus, epidemic, psychological status, mental health, PTSD

## Abstract

**Introduction:** The current outbreak of the novel coronavirus disease 2019 (COVID-19), originating from Wuhan (Hubei, China), has rapidly spread across China and several other countries. During the outbreak of COVID-19, mental health of the general population in Hubei province may be affected. This study aimed to assess the psychological status and associated risk factors of the general population in Hubei province during the COVID-19 outbreak.

**Methods:** A cross-sectional online survey was used to evaluate the symptoms of posttraumatic stress disorder (PTSD), depression, and anxiety, which were assessed by the Chinese version of the Impact of Event Scale—Revised, the Patient Health Questionnaire 9, and the seven-item Generalized Anxiety Disorder Scale, respectively. Coping style was assessed by the Simplified Coping Style Questionnaire. Multivariate logistic regression analysis was carried out to detect factors associated with mental health outcomes.

**Results:** Among 9,225 participants, 44.5% rated symptoms of PTSD, and 17.9 and 12.7% suffered from moderate and severe symptoms of depression and anxiety, respectively. Individuals who were geographically located in Wuhan and familiar with someone who has COVID-19 had more severe symptoms of PTSD, depression, and anxiety, as well as a higher score in passive coping style (*P* < 0.05). Multivariate logistic regression analysis showed that people who were geographically located in Wuhan [odds ratio (OR) = 1.25, 95% confidence interval (CI) = 1.14–1.36, *P* < 0.001] were associated with severe symptoms of PTSD. Besides, individuals who were familiar with someone who had COVID-19 (OR = 2.33, 95% CI = 2.07–2.63, *P* < 0.001; OR = 1.90, 95% CI = 1.66–2.17, *P* < 0.001; OR = 2.06, 95% CI = 1.78–2.39, *P* < 0.001) and had a higher score in passive coping style (OR = 1.16, 95% CI = 1.14–1.17, *P* < 0.001; OR = 1.17, 95% CI = 1.15–1.19, *P* < 0.001; OR = 1.17, 95% CI = 1.15–1.19, *P* < 0.001) were associated with severe symptoms of PTSD, depression, and anxiety. Moreover, a higher score in active coping style (OR = 0.96, 95% CI = 0.95–0.97, *P* < 0.001; OR = 0.94, 95% CI = 0.93–0.94, *P* < 0.001; OR = 0.95, 95% CI = 0.94–0.96, *P* < 0.001) was associated with a lower risk of symptoms of PTSD, depression, and anxiety.

**Conclusions:** During the midphase of COVID-19 outbreak, quite a few people have mental health problems; nearly half of the respondents rated symptoms of PTSD, and approximately one-fifth reported moderate to severe symptoms of anxiety and depression. Our findings may lead to better comprehend the psychological status of the general public and alleviate the public mental health crisis during the COVID-19 outbreak.

## Introduction

The current outbreak of the novel coronavirus disease 2019 (COVID-19), originating from Wuhan (Hubei, China), has rapidly spread across China and several other countries. On March 11, 2020, the World Health Organization announced the COVID-2019 outbreak as a pandemic. To date, the number of deaths associated with COVID-19 significantly exceeds those of the other two coronaviruses [severe acute respiratory syndrome coronavirus (SARS-CoV) and Middle East respiratory syndrome coronavirus (MERS-CoV)], and the outbreak is still ongoing, posing a significant threat to global public health and economy (1).

Infectious outbreak naturally causes profound fear and panic in the society. As a result of rapidly increasing numbers of confirmed COVID-19 cases, patients, hospital staff, and the public have experienced psychological problems, such as anxiety, depression, and stress (2, 3). During the SARS outbreak, several scholars psychologically investigated patients, hospital staff, and noninfected community and reported significant rates of psychiatric and posttraumatic morbidities (2, 3). The MindSpot Clinic (Sydney, Australia) demonstrated a significantly increased number of cases with severe anxiety and depression symptoms during the COVID-19 pandemic (4). A US county-level census pointed out that approximately 33% of rural counties are highly susceptible to COVID-19 (5). A survey carried out in India found that since COVID-19 was declared as a pandemic and led to a nationwide blockade, the majority of Indians have experienced mental health disorders (6). In a cross-sectional study of 15,704 German residents, 44.9% reported mild symptoms of generalized anxiety; 14.3% reported symptoms of major depression, and 65.2% reported symptoms of psychological distress (7). From March 27 to April 6, there were 6,509 people in Germany with more than 50% suffering from symptoms of anxiety and depression (8). In addition, the Central People's Government of the People's Republic of China has adopted extreme measures to mitigate the negative consequences of COVID-19 outbreak. On January 23, 2020, the local government of Wuhan announced suspension of public transportation, with closure of airports, railway stations, and highways, in order to avoid disease transmission. Other cities in Hubei province declared similar traffic control measures following Wuhan immediately. On April 8, 2020, China proclaimed to lift the lockdown of Wuhan. Although the Wuhan government has succeeded in bringing the epidemic under control, its widespread has so far had inevitable psychological consequences (9). During the outbreak, mental healthcare of the public who was affected by the 2019-nCoV epidemic in Hubei province has been under addressed, although the National Health Commission of the People's Republic of China released a notification for Emergency Psychological Crisis Intervention for COVID-19 epidemic on January 26, 2020 (10).

To the best of our knowledge, numerous scholars concentrated on the psychological responses to infectious diseases outbreaks, which were conducted on the groups in hospitals, including patients with SARS/MERS (11, 12), medical staff working to combat the illness (e.g., SARS and COVID-19) (13–15), and survivors of SARS epidemic (16). A previous study reported that 104 residents of Wuhan (under mandatory quarantine) had more severe symptoms of posttraumatic stress disorder (PTSD) than 330 residents of Shanghai (without mandatory quarantine) during the COVID-19 outbreak, although the sample size was relatively small (17). Another study investigated the prevalence of psychosocial problems among the general population under the COVID-19 epidemic and found that Hubei province (eight people) had more severe insomnia and stress symptoms than those who lived in areas outside Hubei province (18). Therefore, further data related to psychological status of noninfected general public in Hubei province are required to understand the full psychosocial dimensions of such infectious diseases. Several previous studies have focused on health condition and mortality rate of patients with COVID-19 infection or suspicion, and all have found psychological health problems (19–22). Ran et al. (23) revealed that the prevalence rates of depression, anxiety, and somatization symptoms were 47.1, 31.0, and 45.9%, respectively, among 1770 Chinese citizens during the peak prevalence of COVID-19, but confirmed or suspected cases of COVID-19 were not excluded. The psychological status of general noninfected people in Hubei province has not attracted the attention of researchers. This study is the first large-scale survey concentrated on psychological status (symptoms of PTSD, depression, and anxiety) and coping style of general noninfected population after 1 month of COVID-19 outbreak in Hubei province. We hypothesized that passive coping style and COVID-19–related exposure risks were associated with worse mental health outcomes, and quite a few people have mental health problems such as symptoms of moderate to severe PTSD, depression, and anxiety. This may be significant for government authorities and healthcare professionals to protect mental health of people who are affected by the COVID-19 outbreak worldwide.

## Methods

### Setting and Participants

We used a cross-sectional survey design and anonymous online questionnaires composed of 75 single choices and short-answer questions to evaluate the psychological status of people living in Hubei province during COVID-19 outbreak, from February 28 to March 21. A total of 11,053 questionnaires from the general population of Hubei province were collected. The questionnaires included detailed demographic, COVID-19–related exposure risks, and psychometric scales. A snowball sampling strategy, concentrated on recruiting noninfected people living in Hubei province, was utilized. The online survey was first disseminated to university students, and they were encouraged to share it with others through WeChat public platform and the mainstream media. Every respondent had his/her own IP address, and at the end of the questionnaire, we would check carefully the IP address and delete the questionnaire with the same IP address. This study was approved by the ethics committee of First Affiliated Hospital of Jinan University (Guangzhou, China, approval letter: KY-2020-044) and obtained the informed written consent from all participants. The survey was anonymous, and confidentiality of information was ensured. The minimum sample size required was obtained by using PASS software (http://www.ncss.com/software/pass/procedures/). The prevalence of psychiatric morbidity was 11.7% in Taiwan based on a previous study focused on the SARS outbreak (24). The estimated acceptable margin of error was 0.1. Thus, the width of two-sided confidence interval (CI) was 0.02, and confidence level was 1 – α = 0.95. The study assumed that the effective and qualified of questionnaire were both 90%. Finally, the minimum target sample size was 4,709.

### Survey Instrument

Demographic data were self-reported by participants, including age, sex, level of education, marital status, occupation, and residential location. COVID-19–related exposure risks included whether a participant knew anyone who was suspected or confirmed to have COVID-19 and whether a participant had adequate knowledge about COVID-19 (don't know, know well, very familiar). Here, the Chinese version of the Impact of Event Scale—Revised (IES-R; range, 0–88), the Patient Health Questionnaire 9 (PHQ-9; range, 0-27), the seven-item Generalized Anxiety Disorder Scale (GAD-7; range, 0-21), and the Simplified Coping Style Questionnaire (SCSQ) were used to assess symptoms of PTSD, depression, anxiety, and coping style, respectively (25). IES-R is a 22-item self-report measure intended to investigate subjective PTSD caused by traumatic life events. The standard cutoff score for screening to identify possible PTSD symptoms is 20 (26, 27). PHQ-9 is a 9-question instrument given to patients in a primary care setting to screen for the presence and severity of depression (28, 29). Item 9 of the PHQ-9 is often used to screen depressed patients for suicide risk by evaluating passive thoughts of death or self-injury within the last 2 weeks. GAD-7 is a self-assessment test, which is utilized to assess generalized anxiety disorder. It consists of seven items with high relevance and adopts a 4-point Likert scoring system from 0 to 3 points. The standard cutoff value for moderate and severe anxiety is 10 or greater (30). Additionally, the total scores in PHQ-9 and GAD-7 were interpreted as follows: PHQ-9, normal (0–4), mild (5–9), moderate (10–14), and severe (15–27); GAD-7, normal (0–4), mild (5–9), moderate (10–14), and severe (15–21). SCSQ is a 20-item measure in Chinese culture, which was developed in 1998 based on the Ways of Coping Questionnaire. SCSQ was designed to assess attitudes and actions that individuals would take in the face of life events. Items were classified in two subscales (positive coping style and negative coping style) and rated on a 4-point Likert scale (e.g., 0 = “not take” to 3 = “usually take”). Higher scores indicated greater use of coping strategies. The Chinese version of the IES-R (31), PHQ-9 (32), GAD-7 (33), and SCSQ-20 (34) has been already used in numerous studies in China with satisfactory reliability and validity.

### Statistical Analysis

Data were statistically analyzed by using SPSS 19.0 software (SPSS, Chicago, IL, USA). The significant level was at the rate of α = 0.05, and all tests were two-tailed. The original scores in the IES-R, PHQ-9, GAD-7, and SCSQ-20 were measured for normal distributions by Kolmogorov–Smirnov test (*p* < 0.05) and were not normally distributed and were therefore presented as median with interquartile ranges (IQRs) (15, 35). The demographic characteristics of respondents, each level of symptoms of PTSD, depression, and anxiety were all presented as numbers and percentages. The nonparametric Mann–Whitney *U* test (15, 36) was used between two groups according to geographic location and being familiar with someone who has COVID-19. We hypothesized that respondents who were in Wuhan and familiar with someone who has COVID-19 had more severe symptoms of PTSD, depression, anxiety, and passive coping. The nonparametric Kruskal–Wallis test (15) was applied to compare the symptoms of PTSD, depression, anxiety, active coping, and passive coping between three groups according to knowledge of the epidemic. Sex, age, education level, marital status, and occupation were included as potential confounding variables. In addition, we assumed that being geographically located in Wuhan, being familiar with someone who has COVID-19, and higher level of passive coping style were risk factors for PTSD, depression, and anxiety. To identify potential risk factors for symptoms of PTSD, depression, and anxiety in noninfected respondents, multivariate logistic regression analysis was undertaken, and odds ratios (ORs) and 95% CIs were obtained from logistic regression models. After adjustment for confounding, variables were chosen based on scientifically established associations and our clinical experience, including age, sex, level of education, marital status, occupation, geographical location, knowledge of epidemic, being familiar with someone who has COVID-19, and coping style.

## Results

### Demographic Characteristics

#### Patients' Demographic Characteristics

In the present study, in all 11,053 questionnaires, 396 questionnaires not filled out completely and correctly were excluded, leading to inclusion of 10,657 valid questionnaires with no missing data. Among them, 1,432 questionnaires from individuals with confirmed or suspected COVID-19 were excluded. Finally, 9,225 noninfected cases were enrolled in the statistical analysis. Study subjects' demographic characteristics are shown in Table 1. Among all the participants, the majority of respondents were men (50.7%), aged 26 to 35 years (42.4%), married (66.6%), with high level of education (55.4% with bachelor's degree or greater), geographically located in Wuhan (49.5%), self-employed (30.4%), knew well of the epidemic (49.5%), and were unfamiliar with someone who has COVID-19 (82.1%) (Table 1).

**Table 1:** Demographic characteristics of respondents.

**Characteristic**	**No. (%) (*n* = 9,225)**
**Gender**	
Male	4,674 (50.7)
Female	4,551 (49.3)
**Age (years)**	
<18	367 (4.0)
18–25	2,071 (22.4)
26–35	3,916 (42.4)
36–45	1,772 (19.2)
46–60	867 (9.4)
>60	232 (2.5)
**Marital status**	
Single or divorced or widowed	3,177 (34.4)
Married	6,048 (66.6)
**Education**	
Senior high school or below	4,115 (44.6)
Bachelor's degree or above	5,110 (55.4)
**Geographic location**	
Wuhan	4,570 (49.5)
Ezhou	1,263 (13.7)
Xiangyang	997 (10.8)
Other cities in Hubei	2,395 (26.0)
**Occupation**	
Medical staff	297 (3.2)
Students	1,112 (12.0)
Self-employed	2,803 (30.4)
Farmers	527 (5.7)
Employed	2,400 (26.0)
Unemployed	989 (10.7)
Others	1,097 (11.9)
**Knowledge of the epidemic**	
Don't know much	329 (3.6)
Know well	4,571 (49.5)
Very familiar with	4,325 (46.9)
**Familiar with someone to have COVID-19**	
Yes	1,655 (17.9)
No	7,570 (82.1)
**Relationship with infected patients**	
Man and wife	30 (1.8)
Parents	31 (1.9)
Offspring	8 (0.5)
Brothers and sisters	61 (3.7)
Friends	1,234 (74.5)
Others	291 (17.6)

#### Psychological Status and Coping Style

Of all respondents, 4,105 (44.5%) rated symptoms of PTSD, and 1,652 (17.9%) suffered from moderate or severe symptoms of depression. According to item 9 of the PHQ-9 scale, 780 (8.5%) respondents were considered to have risks of suicide and self-injury. Besides, 1,172 (12.7%) cases suffered from moderate or severe symptoms of anxiety. In contrast to the influence of COVID-19 outbreak, all respondents' coping style assessed by using SCSQ-20 scale revealed median scores of 22.0 (IQR = 16.0–28.0) of active coping style and 10.0 (IQR = 7.0–14.0) of passive coping style. Moreover, individuals who were geographically located in Wuhan had higher scores in IES-R, PHQ-9, GAD-7, active coping, and passive coping compared with those whose geographical locations were in other cities in Hubei province. People who were familiar with someone who has COVID-19 had higher scores in IES-R, PHQ-9, GAD-7, and passive coping. Persons who were very familiar with the COVID-19 epidemic had lower scores in IES-R, PHQ-9, and GAD-7, whereas they had higher scores in active coping and passive coping (Table 2). Men respondents had higher scores in IES-R (*P* = 0.001, χ^2^ = 3.421), PHQ-9 (*P* = 0.001, χ^2^ = 3.263), and passive coping (*P* = 0.009, χ^2^ = 2.626) than female ones. Respondents had significantly different scores in IES-R (*P* < 0.001, *z* = 333.062), PHQ-9 (*P* < 0.001, *z* = 102.991), GAD-7 (*P* < 0.001, *z* = 175.937), and passive coping (*P* < 0.001, *z* = 236.625) in different occupations. Respondents who had other occupations had lower scores in IES-R, PHQ-9, GAD-7, and passive coping compared with medical staff, students, self-employed, farmers, employed, and unemployed. Respondents had significantly different scores in IES-R (*P* < 0.001, *z* = 87.867), PHQ-9 (*P* < 0.001, *z* = 123.395), GAD-7 (*P* < 0.001, *z* = 104.477), and passive coping (*P* < 0.001, *z* = 74.782). Respondents aged 46 to 60 years and older than 60 years had lower scores in IES-R, PHQ-9, GAD-7, and passive coping compared with other age ranges. Individuals who were married had higher scores in IES-R (*P* < 0.001, *z* = 4.342), active coping (*P* < 0.001, *z* = 4.340), and passive coping (*P* < 0.001, *z* = 4.340), whereas they had lower scores in PHQ-9 (*P* < 0.001, *z* = –4.873). Respondents with high level of education had higher scores in active coping (*P* < 0.001, *z* = 7.825) and passive coping (*P* < 0.001, *z* = 4.079). The aforementioned differences were statistically significant (*P* < 0.05) (Supplementary Table 1). In addition, respondents' demographic characteristics who were residents of Wuhan are summarized in Supplementary Table 2, and prevalences of symptoms of PTSD, depression, anxiety, and coping style, particularly for respondents who were residents of Wuhan, are shown in Supplementary Table 3. Among all the respondents who were residents of Wuhan, 4,570 (49.5%) and 2,202 (48.2%) rated symptoms of PTSD. Additionally, 880 (19.3%) rated moderate or severe symptoms of depression, and 636 (13.9%) rated moderate or severe symptoms of anxiety.

**Table 2:** Prevalence of PTSD symptoms, depressive symptoms, anxiety symptoms, and coping style according to respondents.

**Characteristic**	**No. (%)!!break (*n* = 9,225)**	**Total score, median (IQR)**	
Prevalence			
IES-R, PTSD symptoms		16.0 (4.0–32.0)	
<20	5,120 (55.5)		
≥20	4,105 (44.5)		
PHQ-9, depressive symptoms		3.0 (0.0–8.0)	
<10	7,573 (82.1)		
≥10	1,652 (17.9)		
PHQ-9, depressive symptoms			
0–4 (Normal)	5,300 (57.5)		
5–9 (Mild)	2,273 (24.6)		
10–14 (Moderate)	1,078 (11.7)		
15–27 (Severe)	574 (6.2)		
GAD-7, anxiety symptoms		3.0 (0.0–7.0)	
<10	8,053 (87.3)		
≥10	1,172 (12.7)		
GAD-7, anxiety symptoms			
0–4 (Normal)	5,723 (62.0)		
5–9 (Mild)	2,330 (25.3)		
10–14 (Moderate)	951 (10.3)		
15–21 (Severe)	221 (2.4)		
SCSQ-20, coping styles			
Active coping		22.0 (16.0–28.0)	
Passive coping		10.0 (7.0–14.0)	
**Geographic location**	**Median (IQR)**	***p*** **value**	***Z*** **value**
IES-R		<0.001	7.150
Wuhan (n = 4,570)	18.0 (5.0–34.0)		
Other cities in Hubei (n = 4,655)	14.0 (4.0–30.0)		
PHQ-9		<0.001	4.231
Wuhan	4.0 (0.0–8.0)		
Other cities in Hubei	3.0 (0.0–8.0)		
GAD-7		<0.001	4.670
Wuhan	3.0 (0.0–7.0)		
Other cities in Hubei	3.0 (0.0–7.0)		
Active coping		<0.001	3.337
Wuhan	22.0 (16.0–28.0)		
Other cities in Hubei	21.0 (15.0–28.0)		
Passive coping		<0.001	4.775
Wuhan	11.0 (7.0–14.0)		
Other cities in Hubei	10.0 (6.0–14.0)		
**Familiar with someone to have COVID-19**	**Median (IQR)**	***p*** **value**	***Z*** **value**
IES-R		<0.001	20.071
Yes (n = 1,655)	27.0 (12.0–40.0)		
No (n = 7,570)	14.0 (4.0–29.0)		
PHQ-9		<0.001	16.688
Yes	6.0 (2.0–10.0)		
No	3.0 (0.0–7.0)		
GAD-7		<0.001	18.911
Yes	5.0 (2.0–9.0)		
No	2.0 (0.0–6.0)		
Passive coping		<0.001	8.540
Yes	11.0 (8.0–15.0)		
No	10.0 (7.0–14.0)		
**Knowledge of the epidemic**	**Median (IQR)**	***p*** **value**	***χ***^**2**^ **value**
IES-R		0.011	9.068
Don't know much!!break (*n* = 329)	16.0 (4.0–32.0)		
Know well!!break (*n* = 4,571)	17.0 (5.0–32.0)		
Very familiar with!!break (*n* = 4,325)	15.0 (4.0–32.0)		
PHQ-9		<0.001	68.600
Don't know much	4.0 (0.0–8.0)		
Know well	4.0 (0.0–8.0)		
Very familiar with	3.0 (0.0–7.0)		
GAD-7		<0.001	42.832
Don't know much	2.0 (0.0–7.0)		
Know well	3.0 (0.0–7.0)		
Very familiar with	2.0 (0.0–6.0)		
Active coping		<0.001	358.361
Don't know much	17.0 (11.0–21.0)		
Know well	21.0 (15.0–26.0)		
Very familiar with	24.0 (17.0–30.0)		
Passive coping		<0.001	81.125
Don't know much	9.0 (6.0–13.0)		
Know well	10.0 (7.0–13.0)		
Very familiar with	11.0 (7.0–15.0)		

#### Risk Factors for Symptoms of PTSD, Depression, and Anxiety

According to the results of multivariate logistic regression analysis, after adjusting for other confounding including sex, age, education level, marital status, and occupation, individuals who were geographically located in Wuhan (OR = 1.25, 95% CI = 1.14–1.36, *P* < 0.001) were found to be associated with severe symptoms of PTSD. Individuals who were familiar with someone who has COVID-19 were associated with severe symptoms of PTSD, depression, and anxiety (OR = 2.33, 95% CI = 2.07–2.63, *P* < 0.001; OR = 1.90, 95% CI = 1.66–2.17, *P* < 0.001; OR = 2.06, 95% CI = 1.78–2.39, *P* < 0.001). Compared with not knowing much of the COVID-19 epidemic, those who were very familiar with the COVID-19 outbreak were associated with a lower risk of PTSD symptoms (OR = 0.76, 95% CI = 0.59–0.97, *P* = 0.030). As for coping style, a higher level of active coping style (OR = 0.96, 95% CI = 0.95–0.97, *P* < 0.001; OR = 0.94, 95% CI = 0.93–0.94, *P* < 0.001; OR = 0.95, 95% CI = 0.94–0.96, *P* < 0.001) was associated with a lower risk of symptoms of PTSD, depression, and anxiety. On the contrary, higher level of passive coping style (OR = 1.16, 95% CI = 1.14–1.17, *P* < 0.001; OR = 1.17, 95% CI = 1.15–1.19, *P* < 0.001; OR = 1.17, 95% CI = 1.15–1.19, *P* < 0.001) was associated with severe symptoms of PTSD, depression, and anxiety. Compared with those younger than 18 years, ages 18–25, 26–35, and 36–45 years were significantly associated with severe symptoms of PTSD (OR = 1.60, 95% CI = 1.20–2.14, *P* = 0.001; OR = 1.39, 95% CI = 1.02–1.90, *P* = 0.040; OR = 1.59, 95% CI = 1.15–2.20, *P* = 0.005), and ages older than 60 years were linked with a lower risk of symptoms of depression and anxiety (OR = 0.41, 95% CI = 0.21–0.82, *P* = 0.011; OR = 0.38, 95% CI = 0.18–0.82, *P* = 0.013). Compared with those with bachelor's degree or greater, cases who were at senior high school level or below were associated with severe symptoms of PTSD, depression, and anxiety (OR = 1.25, 95% CI = 1.13–1.38, *P* < 0.001; OR = 1.28, 95% CI = 1.13–1.45, *P* < 0.001; OR = 1.33, 95% CI = 1.15–1.53, *P* < 0.001). Compared with unemployed individuals, students were associated with a lower risk of symptoms of PTSD and anxiety (OR = 0.69, 95% CI = 0.55–0.88, *P* = 0.003; OR = 0.62, 95% CI = 0.44–0.87, *P* = 0.005). Additionally, having other professions was associated with a lower risk of symptoms of PTSD, depressive, and anxiety (OR = 0.58, 95% CI = 0.47–0.71, *P* < 0.001; OR = 0.60, 95% CI = 0.45–0.80, *P* < 0.001; OR = 0.51, 95% CI = 0.37–0.71, *P* < 0.001) (Table 3).

**Table 3:** Results of multivariate logistic regression analyses.

**Variable**	**No. of severe cases/no. of total cases (%)**	**B**	**Standard error**	**Wald**	***P* value**	**OR (95% CI)**
**IES-R, PTSD symptoms**						
Constant	NA	−1.58	0.23	48.08	<0.001^***^	NA
**Geographic location**						
Wuhan	2,202/4,570 (48.2)	0.22	0.05	22.91	<0.001^***^	1.25 (1.14–1.36)
Other cities in Hubei	1,903/4,655 (40.9)	NA	NA	NA	NA	1 [Reference]
**Familiar with someone to have COVID-19**						
Yes	1,052/1,655 (63.6)	0.85	0.06	193.19	<0.001^***^	2.33 (2.07–2.63)
No	3,053/7,570 (40.3)	NA	NA	NA	NA	1 [Reference]
Active coping	NA	−0.04	0.01	124.74	<0.001^***^	0.96 (0.95–0.97)
Passive coping	NA	0.15	0.01	738.88	<0.001^***^	1.16 (1.14–1.17)
**Age (years)**						
<18	107/367 (29.2)	NA	NA	NA	NA	1 [Reference]
18–25	954/2,071 (46.1)	0.47	0.15	10.305	0.001^**^	1.60 (1.20–2.14)
26–35	1,812/3,916 (46.3)	0.33	0.16	4.23	0.040^*^	1.39 (1.02–1.90)
36–45	847/1,772 (47.8)	0.47	0.17	7.87	0.005^**^	1.59 (1.15–2.20)
46–60	315/867 (36.3)	0.15	0.18	0.76	0.382	1.17 (0.83–1.64)
>60	70/232 (30.2)	−0.15	0.23	0.43	0.514	0.86 (0.55–1.35)
**Education**						
Senior high school or below	1,879/4,115 (45.7)	0.23	0.05	19.54	<0.001^***^	1.25 (1.13–1.38)
Bachelor's degree or above	2,226/5,110 (43.6)	NA	NA	NA	NA	1 [Reference]
**Occupation**						
Medical staff	132/297 (44.4)	−0.02	0.15	0.01	0.911	0.98 (0.73–1.32)
Students	382/1,112 (34.4)	−0.37	0.12	9.05	0.003^**^	0.69 (0.55–0.88)
Self-employed	1,439/2,803 (51.3)	0.15	0.09	2.84	0.092	1.17 (0.98–1.39)
Farmers	283/527 (53.7)	0.19	0.12	2.41	0.120	1.21 (0.95–1.54)
Employed	1,173/2,400 (48.9)	0.16	0.09	2.94	0.086	1.17 (0.98–1.40)
Unemployed	394/989 (39.8)	NA	NA	NA	NA	1 [Reference]
Others	302/1,097 (27.5)	−0.55	0.11	26.85	<0.001^***^	0.58 (0.47–0.71)
**Knowledge of the epidemic**						
Don't know much	150/329 (45.6)	NA	NA	NA	NA	1 [Reference]
Know well	2,108/4,571 (46.1)	0.08	0.13	0.36	0.549	1.08 (0.84–1.38)
Very familiar with	1,847/4,325 (42.7)	−0.28	0.13	4.68	0.030^*^	0.76 (0.59–0.97)
**PHQ-9, depressive symptoms**						
Constant	NA	−2.40	0.29	70.77	<0.001^***^	NA
**Familiar with someone to have COVID-19**						
Yes	464/1,655 (28.0)	0.64	0.07	88.89	<0.001^***^	1.90 (1.66–2.17)
No	1,188/7,570 (15.7)	NA	NA	NA	NA	1 [Reference]
Active coping	NA	−0.07	0.01	204.57	<0.001^***^	0.94 (0.93–0.94)
Passive coping	NA	0.16	0.01	494.50	<0.001^***^	1.17 (1.15–1.19)
**Age**						
<18	53/367 (14.4)	NA	NA	NA	NA	1 [Reference]
18–25	440/2,071 (21.2)	0.34	0.19	3.39	0.065	1.41 (0.98–2.02)
26–35	732/3,916 (18.7)	0.21	0.20	1.06	0.304	1.23 (0.83–1.82)
36–45	316/1,772 (17.8)	0.22	0.21	1.10	0.295	1.24 (0.83–1.86)
46–60	96/867 (11.1)	−0.19	0.23	0.68	0.410	0.83 (0.53–1.29)
>60	15/232 (6.5)	−0.88	0.35	6.53	0.011^*^	0.41 (0.21–0.82)
**Education**						
Senior high school or below	788/4,115 (19.1)	0.25	0.06	15.12	<0.001^***^	1.28 (1.13–1.45)
Bachelor's degree or above	864/5,110 (16.9)	NA	NA	‘NA	NA	1 [Reference]
**Occupation**						
Medical staff	47/297 (15.8)	−0.19	0.20	0.95	0.331	0.82 (0.56–1.22)
Students	183/1,112 (16.5)	−0.23	0.15	2.28	0.131	0.79 (0.59–1.22)
Self-employed	611/2,803 (21.8)	0.07	0.12	0.38	0.540	1.07 (0.86–1.35)
Farmers	123/527 (23.3)	0.09	0.15	0.38	0.540	1.10 (0.82–1.47)
Employed	437/2,400 (18.2)	−0.04	0.12	0.11	0.742	0.96 (0.76–1.21)
Unemployed	146/989 (14.8)	NA	NA	NA	NA	1 [Reference]
Others	105/1,097 (9.6)	−0.52	0.15	12.44	<0.001^***^	0.60 (0.45–0.80)
**GAD-7, anxiety symptoms**						
Constant	NA	−2.85	0.32	77.61	<0.001^***^	NA
**Familiar with someone to have COVID-19**						
Yes	353/1,655 (21.3)	0.72	0.08	91.72	<0.001^***^	2.06 (1.78–2.39)
No	819/7,570 (10.8)	NA	NA	NA	NA	1 [Reference]
Active coping	NA	−0.05	0.01	79.19	<0.001^***^	0.95 (0.94–0.96)
Passive coping	NA	0.16	0.01	395.13	<0.001^***^	1.17 (1.15–1.19)
**Age**						
<18	38/367 (10.4)	NA	NA	NA	NA	1 [Reference]
18–25	314/2,071 (15.2)	0.28	0.21	1.68	0.196	1.32 (0.87–2.00)
26–35	515/3,916 (13.2)	0.10	0.23	0.20	0.659	1.11 (0.71–1.73)
36–45	225/1,772 (12.7)	0.12	0.24	0.26	0.607	1.13 (0.71–1.80)
46–60	68/867 (7.8)	−0.35	0.26	1.82	0.177	0.70 (0.42–1.17)
>60	12/232 (5.2)	−0.96	0.39	6.12	0.013^*^	0.38 (0.18–0.82)
**Education**						
Senior high school or below	571/4,115 (13.9)	0.28	0.07	15.70	<0.001^***^	1.33 (1.15–1.53)
Bachelor's degree or above	601/5,110 (11.8)	NA	NA	NA	NA	1 [Reference]
**Occupation**						
Medical staff	35/297 (11.8)	−0.35	0.22	2.50	0.114	0.70 (0.46–1.09)
Students	125/1,112 (11.2)	−0.48	0.17	7.73	0.005^**^	0.62 (0.44–0.87)
Self-employed	422/2,803 (15.1)	−0.18	0.13	2.03	0.154	0.83 (0.65–1.07)
Farmers	90/527 (17.1)	−0.10	0.17	0.33	0.563	0.91 (0.66–1.26)
Employed	308/2,400 (12.8)	−0.25	0.13	3.53	0.060	0.78 (0.61–1.01)
Unemployed	119/989 (12.0)	NA	NA	NA	NA	1 [Reference]
Others	73/1,097 (6.7)	−0.67	0.17	16.14	<0.001^***^	0.51 (0.37–0.71)

* *p < 0.05*,

** *p < 0.01*,

*** *p < 0.001*.

## Discussion

The findings of the present survey suggest initial psychological responses of noninfected individuals living in Hubei province from February 28 to March 21. About 6 weeks after the COVID-19 outbreak, the Wuhan government imposed an unprecedented extensive blockade for 5 weeks and indefinite traffic restrictions. The results unveiled that 44.5% of respondents rated the PTSD symptoms, 17.9% of respondents reported moderate to severe depressive symptoms, and 12.7% of respondents reported moderate to severe anxiety symptoms. People who were geographically located in Wuhan and those who were familiar with someone who has COVID-19 reported more severe symptoms of PTSD, depression, and anxiety. Moreover, passive coping style and being familiar with someone who has COVID-19 were found to be associated with worse mental health outcomes. To our knowledge, this is the first large sample survey concentrated on individuals' psychological status living in Hubei province since the outbreak of COVID-19.

Our results showed that a substantial proportion of residents of Hubei province, especially residents of Wuhan, had PTSD, as evidenced by the proportion of symptoms of PTSD, depression, and anxiety. Similarly, more than half of the participants felt helpless because of the COVID-19 pandemic, and a mild stressful impact was found on local Chinese residents in Liaoning province (37). The prevalence of symptoms of anxiety and depression was in agreement with that reported in the outbreak of SARS and MERS and during the initial stage of the COVID-19 epidemic among the general population in China (26, 30, 38). However, the prevalence of PTSD symptoms in the current study was greater than that reported during the outbreak of SARS and MERS (26, 30, 39). The following reasons might account for this phenomenon: (1) official confirmation of human-to-human transmission of COVID-19; (2) the local government of Wuhan imposed unprecedented widespread lockdown and traffic restrictions, and similar measures were adopted in other cities in Hubei province; (3) lack of medical protection resources in the early stage of the COVID-19 epidemic; and (4) Wuhan is the center of the outbreak, with the greatest number of people infected, the most exposed information, and the stronger impact on people's emotions. Furthermore, the present study was carried out at 6 weeks after the COVID-19 outbreak and 5 weeks after the blockade and traffic restrictions, which were different from the initial stage of the epidemic (38, 40). Over the past month and a half, people have gone through an adaption process that better reflects the profound impact of the epidemic on their psychological responses. Moreover, individuals who knew their family and friends to have COVID-19 had more severe symptoms of PTSD, depression, and anxiety. Such people were likely at a high risk of infection because of their close and frequent contact with COVID-19 patients and may warrant early and focused support services. Although persons underwent symptoms suggestive of depression, anxiety, and PTSD, the scales that were used to evaluate these symptoms were noted insufficient to confirm these diagnoses. Hence, further structured diagnostic interviews are required to confirm a diagnosis of depression, anxiety, and PTSD.

Coping style can be divided into active coping and passive coping. Active coping refers to taking a direct and rational way to solve a problem, whereas passive coping is linked to dealing with problems by avoidance, withdrawal, and denial (41). Fu et al. found 70.2% of Wuhan residents adopted active coping style, such as taking part in activities, talking to others, and maintaining an optimistic attitude, but 29.8% relied on passive coping style during the outbreaks (42). In the current study, a higher level of passive coping style was associated with severe symptoms of PTSD, depression, and anxiety, whereas a higher level of active coping style was associated with a lower risk of psychological symptoms. These findings indicated that more passive coping and less active coping style were risk factors for worse mental health outcomes. Previous studies demonstrated that passive coping could be an important risk factor for PTSD, affective disorders, and suicide (43–45). A number of scholars pointed out that active coping–based strategies were conducive to positive psychosocial outcomes (46, 47). In addition, studies emphasized that coping style–based methods could mediate the relationship between social support and individuals' adjustment outcomes, including psychological distress and depression (48). Taken together, the aforementioned results highlighted the importance of integrating coping style–based methods into psychological interventions during the COVID-19 epidemic.

As the COVID-19 epidemic continues to spread, our findings may provide vital guidance for the improvement of public mental health strategies: (1) health authorities need to pay further attention to high-risk groups based on social demographic information such as geographic location in Wuhan, being familiar with someone who has COVID-19, being at senior high school level or below, and unemployed individuals for early psychological interventions; (2) health authorities need to identify immediate psychological needs of general population who develops worse mental health outcomes during the epidemic; (3) the government and health authorities should urgently provide accurate data during the epidemic to reduce the impact of rumors; (4) promotion of positive coping style–based strategies is highly encouraged to support the needs of general population during the epidemic; (5) secure services should be set up to provide psychological counseling using electronic devices and applications (e.g., smartphones and tablets) for affected patients, as well as their families and members of the public; and (6) integrated crisis prevention and intervention systems, including epidemiological surveillance, screening, referral, and targeted interventions, should be provided to reduce symptoms of PTSD and prevent further mental health problems.

This timely survey on the psychological status and coping styles of general populations during the COVID-19 epidemic included 9,225 respondents in Hubei province, a sample size larger than that of most related studies. Although Hubei province is the origin of the epidemic, the general populations in other provinces may have similar psychological conditions as a result of COVID-19. In addition, a comparative study on the psychological status of the general population in Hubei before and after the blockade can be compared in the future. However, this study has several limitations. First, we adopted snowball sampling strategy. The snowball sampling strategy is not based on random selection of samples and does not truly reflect the actual pattern of the general population. Second, a self-selection effect might have occurred for those individuals who experienced the greatest or least levels of PTSD. Third, lack of household income information in the questionnaire made it infeasible to assess the impact of income on mental health. Fourth, this was a cross-sectional study that examined respondents' psychological status, and it could not determine whether respondents' psychological status was affected by the COVID-19 epidemic. Fifth, although we found that having other occupations was markedly associated with a lower risk of symptoms of PTSD, depression, and anxiety compared with unemployed individuals, the questionnaire did not provide details on other occupations. Finally, respondents had to use a computer or smartphone to respond, suggesting that they may be more educated and socioeconomically stable than the population as a whole.

## Conclusions

During the midphase of the COVID-19 outbreak in Hubei province, nearly half of the respondents rated PTSD symptoms, and approximately one-fifth reported moderate and severe symptoms of anxiety and depression. Moreover, passive coping style and COVID-19–related exposure risks were considered to be associated with worse mental health outcomes. Therefore, it is highly essential to establish early practical public mental health programs for population in places where the epidemic originated, so as to improve the mental health and quality of life of affected population.

## Data Availability Statement

The original contributions presented in the study are included in the article/Supplementary Material, further inquiries can be directed to the corresponding author/s.

## Ethics Statement

The studies involving human participants were reviewed and approved by the Ethics Committee of First Affiliated Hospital of Jinan University (Guangzhou, China, Approval Letter: KY-2020-044). The patients/participants provided their written informed consent to participate in this study.

## Author Contributions

YW and LH: design the study. GC, JG, ZQ, SZ, TS, and JW: contribute to data acquisition. GC and YW: contribute to data analysis. GC, JG, and ZQ: write the manuscript. YW and LH: revise the manuscript. All authors contribute to and have approved the final manuscript.

## Conflict of Interest

The authors declare that the research was conducted in the absence of any commercial or financial relationships that could be construed as a potential conflict of interest.
