# Supplementary material for: The Psychological Status of General Population in Hubei Province During the COVID-19 Outbreak: A Cross-Sectional Survey Study
Source: Front Public Health. 2021 Apr 22;9:622762. doi: 10.3389/fpubh.2021.622762 (PMC8100308; doi:10.3389/fpubh.2021.622762)
Supplement: Supplementary file 1 [file Table_1.DOCX]

**Supplementary material**

**Table S1.** Psychological status and coping style according to respondents demographics.

|  | **IES-R, PTSD symptoms** | | | **PHQ-9, depressive symptoms** | | | **GAD-7, anxiety symptoms** | | | **Active coping** | | | **Passive coping** | | |
| --- | --- | --- | --- | --- | --- | --- | --- | --- | --- | --- | --- | --- | --- | --- | --- |
|  | **Median (IQR)** | ***p*-value** | ***Z/X^2^* value** | **Median (IQR)** | ***p*-value** | ***Z/X^2^* value** | **Median (IQR)** | ***p*-value** | ***Z/X^2^* value** | **Median (IQR)** | ***p*-value** | ***Z/X^2^* value** | **Median (IQR)** | ***p*-value** | ***Z/X^2^* value** |
| **Gender** |  | 0.001^*^ | 3.421 |  | 0.001^*^ | 3.263 |  |  |  |  | 0.001^*^ | −3.187 |  | 0.009^*^ | 2.626 |
| Male (*n* = 4,674) | 17.0 (4.0–33.0) |  |  | 4.0 (0.0–8.0) |  |  |  |  |  | 21.0 (16.0–27.0) |  |  | 11.0 (7.0–14.0) |  |  |
| Female (*n* = 4,551) | 15.0 (4.0–30.0) |  |  | 3.0 (0.0–8.0) |  |  |  |  |  | 22.0 (16.0–28.0) |  |  | 10.0 (7.0–13.0) |  |  |
| **Age** |  | <0.001^**^ | 87.867 |  | <0.001^**^ | 123.395 |  | <0.001^**^ | 104.477 |  | <0.001^**^ | 43.314 |  | <0.001^**^ | 74.782 |
| ＜18 (*n* = 367) | 9.0 (2.0–22.0) |  |  | 3.0 (0.0–8.0) |  |  | 1.0 (0.0–5.0) |  |  | 20.0 (14.0–27.0) |  |  | 9.0 (5.0–13.0) |  |  |
| 18–25 (*n* = 2,071) | 17.0 (4.0–33.0) |  |  | 4.0 (1.0–9.0) |  |  | 3.0 (0.0–7.0) |  |  | 21.0 (16.0–27.0) |  |  | 11.0 (7.0–14.0) |  |  |
| 26–35 (*n* = 3916) | 17.0 (4.0–33.0) |  |  | 3.0 (0.0–8.0) |  |  | 3.0 (0.0–7.0) |  |  | 22.0 (16.0–28.0) |  |  | 11.0 (7.0–14.0) |  |  |
| 36–45 (*n* = 1,772) | 18.0 (5.0–33.0) |  |  | 3.0 (0.0–8.0) |  |  | 3.0 (0.0–7.0) |  |  | 22.0 (16.0–27.0) |  |  | 10.0 (7.0–14.0) |  |  |
| 46–60 (*n* = 867) | 13.0 (4.0–25.0) |  |  | 2.0 (0.0–6.0) |  |  | 1.0 (0.0–5.0) |  |  | 23.0 (17.0–29.0) |  |  | 10.0 (6.0–13.0) |  |  |
| 60+ (*n =* 232) | 13.0 (5.0–23.0) |  |  | 1.0 (0.0–4.0) |  |  | 1.0 (0.0–5.0) |  |  | 21.0 (13.0–27.0) |  |  | 9.0 (5.0–13.0) |  |  |
| **Marital status** |  | <0.001^**^ | −4.342 |  | <0.001^**^ | 4.873 |  |  |  |  | <0.001^**^ | −4.340 |  | 0.002^*^ | −3.054 |
| Single or divorced or widowed (*n =* 3177) | 14.0 (4.0–30.0) |  |  | 4.0 (0.0–8.0) |  |  |  |  |  | 21.0 (16.0–27.0) |  |  | 10.0 (6.0–14.0) |  |  |
| Married ( *n =* 6048) | 17.0 (5.0–33.0) |  |  | 3.0 (0.0–8.0) |  |  |  |  |  | 22.0 (16.0–28.0) |  |  | 11.0 (7.0–14.0) |  |  |
| **Education** |  |  |  |  |  |  |  |  |  |  | <0.001^**^ | −7.825 |  | <0.001^**^ | −4.079 |
| Senior high school or below |  |  |  |  |  |  |  |  |  | 21.0 (15.0–27.0) |  |  | 10.0 (6.0–14.0) |  |  |
| Bachelor’s degree or above |  |  |  |  |  |  |  |  |  | 22.0 (17.0–28.0) |  |  | 11.0 (7.0–14.0) |  |  |
| **Occupation** |  | <0.001^**^ | 333.062 |  | <0.001^**^ | 102.991 |  | <0.001^**^ | 175.937 |  | <0.001^**^ | 31.671 |  | <0.001^**^ | 236.625 |
| Medical staff (*n =* 297) | 15.0 (4.0–33.5) |  |  | 3.0 (0.0–8.0) |  |  | 2.0 (0.0–7.0) |  |  | 23.0 (17.0–29.0) |  |  | 10.0 (6.5–14.0) |  |  |
| Students (*n =* 1112) | 12.0 (3.0–25.0) |  |  | 3.0 (0.0–8.0) |  |  | 2.0 (0.0–6.0) |  |  | 21.0 (16.0–27.0) |  |  | 10.0 (6.0–13.0) |  |  |
| Self–employed (*n =* 2803) | 20.0 (6.0–35.0) |  |  | 4.0 (0.0–9.0) |  |  | 3.0 (0.0–7.0) |  |  | 22.0 (16.0–28.0) |  |  | 11.0 (8.0–15.0) |  |  |
| Farmers (*n =* 527) | 22.0 (7.0–36.0) |  |  | 4.0 (0.0–9.0) |  |  | 4.0 (0.0–8.0) |  |  | 20.0 (15.0–27.0) |  |  | 11.0 (8.0–14.0) |  |  |
| Employed (*n =* 2,400) | 19.0 (6.0–33.0) |  |  | 4.0 (0.0–8.0) |  |  | 3.0 (0.0–7.0) |  |  | 22.0 (17.0–28.0) |  |  | 11.0 (8.0–14.0) |  |  |
| Unemployed (*n =* 989) | 15.0 (5.0–28.5) |  |  | 3.0 (0.0–7.5) |  |  | 2.0 (0.0–7.0) |  |  | 21.0 (15.0–27.0) |  |  | 10.0 (6.0–13.0) |  |  |
| Others (*n =* 1,097) | 7.0 (2.0–21.0) |  |  | 2.0 (0.0–6.0) |  |  | 1.0 (0.0–5.0) |  |  | 22.0 (15.0–28.0) |  |  | 8.0 (5.0–12.0) |  |  |
| ^*^*p*<0.01; ^**^ *p*<0.001 |  |  |  |  |  |  |  |  |  |  |  |  |  |  |  |

**Table S2. Demographic characteristics of respondents in Wuhan.**

| **Characteristic** | **No. (%) (***N =* **4570)** |
| --- | --- |
| **Gender** |  |
| Male | 2487 (54.4) |
| Female | 2083 (45.6) |
| **Age** |  |
| ＜18 | 167 (3.7) |
| 18-25 | 1137 (24.9) |
| 26-35 | 1956 (42.8) |
| 36-45 | 898 (19.6) |
| 46-60 | 334 (7.3) |
| 60+ | 78 (1.7) |
| **Marital status** |  |
| Single or divorced or widowed | 1634 (35.8) |
| Married | 2936 (64.2) |
| **Education** |  |
| Senior high school or below | 1854 (40.6) |
| Bachelor’s degree or above | 2716 (59.4) |
| **Geographic location** |  |
| Wuhan | 4570 (100) |
| Other cities in Hubei | 0 (NA) |
| **Occupation** |  |
| Medical staff | 178 (3.9) |
| Students | 561 (12.3) |
| Self-employed | 1472 (32.2) |
| Farmers | 246 (5.4) |
| Employed | 1202 (26.3) |
| Unemployed | 429 (9.4) |
| Others | 482 (10.5) |
| **Knowledge of the epidemic** |  |
| Don't know much | 177 (3.9) |
| Know well | 2153 (47.1) |
| Very familiar with | 2240 (49.0) |
| **Familiar with someone to have COVID-19** |  |
| Yes | 985 (21.6) |
| No | 3585 (78.4) |
| **Relationship with infected patients** |  |
| Man and wife | 20 (2.0) |
| Parents | 24 (2.4) |
| Offsprings | 5 (0.5) |
| Brothers and sisters | 43 (4.4) |
| Friends | 738 (74.9) |
| Others | 155 (15.7) |

**Table S3. Prevalence of PTSD symptoms, depressive symptoms, anxiety symptoms, and coping style according to respondents in Wuhan**

| **Characteristic** | **No. (%) (*N* = 4570)** | **Total score, median (IQR)** |
| --- | --- | --- |
| Prevalence |  |  |
| IES-R, PTSD symptoms |  | 18.0 (5.0-34.0) |
| <20 | 2368 (51.8) |  |
| ≥20 | 2202 (48.2) |  |
| PHQ-9, depressive symptoms |  | 4.0 (0.0-8.0) |
| <10 | 3690 (80.7) |  |
| ≥10 | 880 (19.3) |  |
| PHQ-9, depressive symptoms |  |  |
| 0-4 (Normal) | 2564 (56.1) |  |
| 5-9 (Mild) | 1126 (24.6) |  |
| 10-14 (Moderate) | 576 (12.6) |  |
| 15-27 (Severe) | 304 (6.7) |  |
| GAD-7, anxiety symptoms |  | 3.0 (0.0-7.0) |
| <10 | 3934 (86.1) |  |
| ≥10 | 636 (13.9) |  |
| GAD-7, anxiety symptoms |  |  |
| 0-4 (Normal) | 2723 (59.6) |  |
| 5-9 (Mild) | 1211 (26.5) |  |
| 10-14 (Moderate) | 520 (11.4) |  |
| 15-21 (Severe) | 116 (2.5) |  |
| SCSQ-20, coping styles |  |  |
| Active coping |  | 22.0 (16.0-28.0) |
| Passive coping |  | 11.0 (7.0-14.0) |

**Table S4. Prevalence of PTSD symptoms, depressive symptoms, anxiety symptoms, and coping style according to respondents in Wuhan**

| **Characteristic** | **No. (%) (*N =* 4570)** | **Total score, median (IQR)** |
| --- | --- | --- |
| Prevalence |  |  |
| IES-R, PTSD symptoms |  | 18.0 (5.0-34.0) |
| <20 | 2368 (51.8) |  |
| ≥20 | 2202 (48.2) |  |
| PHQ-9, depressive symptoms |  | 4.0 (0.0-8.0) |
| <10 | 3690 (80.7) |  |
| ≥10 | 880 (19.3) |  |
| PHQ-9, depressive symptoms |  |  |
| 0-4 (Normal) | 2564 (56.1) |  |
| 5-9 (Mild) | 1126 (24.6) |  |
| 10-14 (Moderate) | 576 (12.6) |  |
| 15-27 (Severe) | 304 (6.7) |  |
| GAD-7, anxiety symptoms |  | 3.0 (0.0-7.0) |
| <10 | 3934 (86.1) |  |
| ≥10 | 636 (13.9) |  |
| GAD-7, anxiety symptoms |  |  |
| 0-4 (Normal) | 2723 (59.6) |  |
| 5-9 (Mild) | 1211 (26.5) |  |
| 10-14 (Moderate) | 520 (11.4) |  |
| 15-21 (Severe) | 116 (2.5) |  |
| SCSQ-20, coping styles |  |  |
| Active coping |  | 22.0 (16.0-28.0) |
| Passive coping |  | 11.0 (7.0-14.0) |

**Appendix:**

Investigation on the physical and mental state of the public in Hubei during COVID-19 outbreak

A1. Your gender is: [Single choice] *

○Male

○Female

A2. Your age is: [Single choice] *

○Under 18 years old

○18-25 years old

○26-35 years old

○36-45 years old

○46-60 years old

○Over 60 years old

A3. Current geographic location [Single choice] *

○Wuhan City

○Ezhou City

○Xiangyang City

○Other cities in Hubei _________________ *

A4. The occupation you are currently engaged in is: [single choice] *

○Medical staff

○Student

○Self-employed

○ Farmer

○Employed

○Unemployed

○Others

A5. Your educational background: [Single choice] *

○Senior high school or below

○ Bachelor’s degree or above

A6. Your marital status: [single choice] *

○Single or divorced or widowed

○Married

A7. Do you know the origin, transmission route, and protection approaches of this epidemic? [Single-choice question] *

○Don’t know much

○Know well

○Very familiar with

A8. What is your own physical condition at present? [Single-choice question] *

○Healthy

○Being under observation at home isolation because a family member/friend is diagnosed or suspected of COVID-19

○Is a suspected patient with COVID-19, under fixed-point isolation

○Is a patient diagnosed with COVID-19 and is being treated in hospital isolation

○Suffering from diseases other than COVID-19

A9. Are there any suspected or confirmed cases of COVID-19 around you [single choice] *

○Yes

○No

A10. The relationship between suspected or confirmed cases of COVID-19 and you: [single choice] *

○Man and wife

○Parents

○Offsprings

○Brothers and sisters

○Friends

○Others _________________ *

In the past two weeks, how long have you been troubled by the following questions?

B1. I'm not interested in doing anything, it's boring [Single choice] *

○Not at all

○ a few days

○More than half of the days

○Almost every day

B2. Feeling low, depressed, hopeless [Single choice] *

○Not at all

○ a few days

○More than half of the days

○Almost every day

B3. Difficulty falling asleep, always awake, or sleeping too much, lethargy [Single choice] *

○Not at all

○ a few days

○More than half of the days

○Almost every day

B4. Often feel very tired or listless [Single choice] *

○Not at all

○ a few days

○More than half of the days

○Almost every day

B5. Poor appetite, or eating too much [Single choice] *

○Not at all

○ a few days

○More than half of the days

○Almost every day

B6. Feeling bad or failing, or letting myself or my family down [Single choice] *

○Not at all

○ a few days

○More than half of the days

○Almost every day

B7. It is difficult to concentrate, such as memory loss when reading newspapers or watching TV [Single choice] *

○Not at all

○ a few days

○More than half of the days

○Almost every day

B8. Move or speak so slowly that others notice, or vice versa, fidget, irritability, and move around [Single choice] *

○Not at all

○ a few days

○More than half of the days

○Almost every day

B9. There are thoughts of dying or hurting yourself in some way [Single choice] *

○Not at all

○ a few days

○More than half of the days

○Almost every day

B10. How much difficulty did these problems cause in your work, family affairs, or getting along with others [Single choice] *

○Not at all

○ a few days

○More than half of the days

○Almost every day

In the past two weeks, how long have you been troubled by the following questions?

C1. Feeling nervous, anxious or irritable [Single choice] *

○Not at all

○ a few days

○More than half of the days

○Almost every day

C2. Can't stop or control worry [Single choice] *

○Not at all

○ a few days

○More than half of the days

○Almost every day

C3. Worrying too much about all kinds of things [Single choice] *

○Not at all

○ a few days

○More than half of the days

○Almost every day

C4. It’s hard to relax [Single choice] *

○Not at all

○ a few days

○More than half of the days

○Almost every day

C5. Unable to sit still due to anxiety [Single choice] *

○Not at all

○ a few days

○More than half of the days

○Almost every day

C6. Becomes irritable or irritable easily [Single choice] *

○Not at all

○ a few days

○More than half of the days

○Almost every day

C7. Fear that something terrible will happen [Single choice] *

○Not at all

○ a few days

○More than half of the days

○Almost every day

Please Read each statement carefully and choose what you feel is most appropriate at this moment. There is no right or wrong answer.

D1. Any publicity and anything related to the COVID-19 epidemic causes me discomfort. [Single-choice question] *

○No

○rarely

○Sometimes

○Often

○Always

D2. It's hard for me to sleep well until dawn. [Single-choice question] *

○No

○rarely

○Sometimes

○Often

○Always

D3. Something else reminds me of COVID-19. [Single-choice question] *

○No

○rarely

○Sometimes

○Often

○Always

D4. I felt I was easily provoked and easily angered. [Single-choice question] *

○No

○rarely

○Sometimes

○Often

○Always

D5. Whenever I think about COVID-19, or anything else that reminds me of it, I try not to upset myself. [Single-choice question] *

○No

○rarely

○Sometimes

○Often

○Always

D6. I can't help but think about COVID-19. [Single-choice question] *

○No

○rarely

○Sometimes

○Often

○Always

D7. It feels like COVID-19 is not real or never happened. [Single-choice question] *

○No

○rarely

○Sometimes

○Often

○Always

D8. I try to stay away from anything that reminds me of COVID-19. [Single-choice question] *

○No

○rarely

○Sometimes

○Often

○Always

D9. Images of COVID-19 pop up in my mind. [Single-choice question] *

○No

○rarely

○Sometimes

○Often

○Always

D10. I feel nervous and easily frightened. [Single-choice question] *

○No

○rarely

○Sometimes

○Often

○Always

Please Read each statement carefully and choose what you feel is most appropriate at this moment. There is no right or wrong answer.

D11. I try not to think about COVID-19. [Single-choice question] *

○No

○rarely

○Sometimes

○Often

○Always

D12. I realize that I still have a lot of feelings about COVID-19, but I don't know how to deal with it. [Single-choice question] *

○No

○rarely

○Sometimes

○Often

○Always

D13. I feel that I'm kind of numb to the COVID-19. [Single-choice question] *

○No

○rarely

○Sometimes

○Often

○Always

D14. I seem to be surrounded by viruses now. [Single-choice question] *

○No

○rarely

○Sometimes

○Often

○Always

D15. I have difficulty falling asleep. [Single-choice question] *

○No

○rarely

○Sometimes

○Often

○Always

D16. I have strong emotional fluctuations due to the COVID-19 pandemic. [Single-choice question] *

○No

○rarely

○Sometimes

○Often

○Always

D17. I want to forget about it. [Single-choice question] *

○No

○rarely

○Sometimes

○Often

○Always

D18. I feel it is difficult to concentrate. [Single-choice question] *

○No

○rarely

○Sometimes

○Often

○Always

D19. Thinking of COVID-19 will cause physical reactions such as sweating, difficulty breathing, nausea, dizziness, and rapid heartbeat. [Single-choice question] *

○No

○rarely

○Sometimes

○Often

○Always

D20. I had a dream about COVID-19. [Single-choice question] *

○No

○rarely

○Sometimes

○Often

○Always

D21. I feel very alert or guarded. [Single-choice question] *

○No

○rarely

○Sometimes

○Often

○Always

D22. I try not to mention COVID-19. [Single-choice question] *

○No

○rarely

○Sometimes

○Often

○Always

Facing the pressure brought by the epidemic, what attitudes and methods are you more inclined to adopt?

E1. Relieve through work, study or some other activities [Single choice] *

○Not taken

○take occasionally

○ sometimes taken

○Take often

E2. Talk to people and talk about inner troubles [Single choice] *

○Not taken

○take occasionally

○ sometimes taken

○Take often

E3. Try to see the good side of things [Single choice] *

○Not taken

○take occasionally

○ sometimes taken

○Take often

E4. Change your mind and rediscover the more important things in life [Single choice] *

○Not taken

○take occasionally

○ sometimes taken

○Take often

E5. Don’t take the problem too seriously [Single choice] *

○Not taken

○take occasionally

○ sometimes taken

○Take often

E6. Stick to your own position and strive for what you want [Single choice] *

○Not taken

○take occasionally

○ sometimes taken

○Take often

E7. Find out a variety of different ways to solve the problem [Single choice] *

○Not taken

○take occasionally

○ sometimes taken

○Take often

E8. Seek advice from relatives, friends or classmates [Single choice] *

○Not taken

○take occasionally

○ sometimes taken

○Take often

E9. Change the original practice [Single choice] *

○Not taken

○take occasionally

○ sometimes taken

○Take often

E10. Learn from others' experience in dealing with similar difficulties [Single choice] *

○Not taken

○take occasionally

○ sometimes taken

○Take often

Facing the pressure brought by the epidemic, which attitudes and methods are you more inclined to adopt?

E11. Seek hobbies and actively participate in cultural and sports activities [Single choice] *

○Not taken

○take occasionally

○ sometimes taken

○Take often

E12. Try to restrain your emotions, such as disappointment, regret, sadness and anger [Single choice] *

○Not taken

○take occasionally

○ sometimes taken

○Take often

E13. Attempt to take a break or vacation, and temporarily put aside the problem (worries) [Single choice] *

○Not taken

○take occasionally

○ sometimes taken

○Take often

E14. Eliminate worries by smoking, drinking, taking medicine and eating [Single choice] *

○Not taken

○take occasionally

○ sometimes taken

○Take often

E15. I think time will change the status quo, I just need to wait [Single choice] *

○Not taken

○take occasionally

○ sometimes taken

○Take often

E16. Trying to forget the whole thing [Single choice] *

○Not taken

○take occasionally

○ sometimes taken

○Take often

E17. Rely on others to solve problems [Single choice] *

○Not taken

○take occasionally

○ sometimes taken

○Take often

E18. Accept the reality, because there is no other way [Single choice] *

○Not taken

○take occasionally

○ sometimes taken

○Take often

E19. Fantasy that some miracle may happen to change the status quo [Single choice] *

○Not taken

○take occasionally

○ sometimes taken

○Take often

E20. Comfort yourself [Single choice] *

○Not taken

○take occasionally

○ sometimes taken

○Take often

If you have questions or suggestions about this survey, please write them down below. [Fill in the blanks]

_________________________________

Name: _________ Age: ___ years old

Phone/WeChat/QQ/E-mail: ____________ [fill in the blanks] *

Please leave your true and accurate contact information, there may be related staff to contact you in the future, I wish you good health.
